# Entry and Elimination of Marine Mammal *Brucella* spp. by Hooded Seal (*Cystophora cristata*) Alveolar Macrophages *In Vitro*

**DOI:** 10.1371/journal.pone.0070186

**Published:** 2013-07-25

**Authors:** Anett K. Larsen, Ingebjørg H. Nymo, Preben Boysen, Morten Tryland, Jacques Godfroid

**Affiliations:** 1 Section for Arctic Veterinary Medicine, Department of Food Safety and Infection Biology, Norwegian School of Veterinary Science, Tromsø, Norway; 2 Member of the Fram Centre, High North Research Centre for Climate and the Environment, Tromsø, Norway; 3 Section for Microbiology, Immunology, and Parasitology, Department of Food Safety and Infection Biology, Norwegian School of Veterinary Science, Oslo, Norway; Institut National de la Recherche Agronomique, France

## Abstract

A high prevalence of *Brucella* *pinnipedialis* serology and bacteriology positive animals has been found in the Northeast Atlantic stock of hooded seal ( *Cystophora* *cristata* ); however no associated gross pathological changes have been identified. Marine mammal brucellae have previously displayed different infection patterns in human and murine macrophages. To investigate if marine mammal *Brucella* spp. are able to invade and multiply in cells originating from a presumed host species, we infected alveolar macrophages from hooded seal with a *B* *. pinnipedialis* hooded seal isolate. Hooded seal alveolar macrophages were also challenged with *B* *. pinnipedialis* reference strain (NCTC 12890) from harbor seal ( *Phoca* *vitulina* ), *B* *. ceti* reference strain (NCTC 12891) from harbor porpoise ( *Phocoena* *phocoena* ) and a *B* *. ceti* Atlantic white-sided dolphin ( *Lagenorhynchus* *acutus* ) isolate (M83/07/1), to evaluate possible species-specific differences. *Brucella suis* 1330 was included as a positive control. Alveolar macrophages were obtained by post mortem bronchoalveolar lavage of euthanized hooded seals. Phenotyping of cells in the lavage fluid was executed by flow cytometry using the surface markers CD14 and CD18. Cultured lavage cells were identified as alveolar macrophages based on morphology, expression of surface markers and phagocytic ability. Alveolar macrophages were challenged with *Brucella* spp. in a gentamicin protection assay. Following infection, cell lysates from different time points were plated and evaluated quantitatively for colony forming units. Intracellular presence of *B* *. pinnipedialis* hooded seal isolate was verified by immunocytochemistry. Our results show that the marine mammal brucellae were able to enter hooded seal alveolar macrophages; however, they did not multiply intracellularly and were eliminated within 48 hours, to the contrary of *B. suis* that showed the classical pattern of a pathogenic strain. In conclusion, none of the four marine mammal strains tested were able to establish a persistent infection in primary alveolar macrophages from hooded seal.

## Introduction

Brucellosis, caused by the facultative intracellular bacteria *Brucella* spp., is a contagious disease known to affect a wide range of animal species, and some members of the genus are also zoonotic. Replication of the organism in the reproductive system in primary hosts is associated with abortion and sterility, and persistence in macrophages causing chronic infections is a hallmark of brucellosis in both primary and secondary hosts [1]. *Brucella* spp. were isolated from marine mammals for the first time in 1994 [2] and validly published as members of the genus *Brucella* with the names *Brucella* *pinnipedialis* (pinnipeds; seals, sea lions and walruses) and *Brucella* *ceti* (cetaceans; whales, dolphins, and porpoises) in 2007 [3]. Marine mammal brucellae have since been serologically indicated in and isolated from pinnipeds and cetaceans from various locations around the world. Gross pathology in association with *Brucella* infection in marine mammals is reported exclusively in cetaceans, mainly in the central nervous system and the reproductive organs [4–6].

The hooded seal ( *Cystophora* *cristata* ) is a pelagic species distributed throughout the North Atlantic Ocean. They comprise two stocks; the Northeast and the Northwest Atlantic, breeding off the eastern and western coast of Greenland, respectively [7]. The Northeast Atlantic stock was approximately 82,400 animals in 2005, only 10–15% of the population estimate in 1946, but has been stable at this low level since the 1980s [8]. In contrast, the Northwest Atlantic stock has increased in recent decades and the size was estimated to be 593,500 animals in 2005 [9]. Both hooded seal stocks have been subjected to commercial exploitation since the late seventeenth century [10], however; during the last 25 years, the hooded seal hunt for both stocks has been regulated by quotas. The concern for the stable yet low Northeast Atlantic stock has led to the closure of hunting of this stock since 2007 [11], and the species has been classified as “vulnerable” in the Red List of Threatened Species of the International Union for Conservation of Nature (IUCN) since 2008 [12]. Previous serological investigations for anti- *Brucella* antibodies in hooded seals have shown seroprevalences of 31–35% in the declined Northeast Atlantic stock [13,14] compared to 5% in the increasing Northwest Atlantic stock [15]. Investigation of 29 apparently healthy young adult hooded seals from the Northeast Atlantic stock revealed isolation of *B* *. pinnipedialis* from 11 of the 29 animals, with the highest tissue prevalence in spleen and lung lymph nodes [14]. *Brucella* *pinnipedialis* has also been isolated from multiple organs in three young hooded seals, stranded on the coast of Scotland, with no *Brucella* -associated pathology [4]. Whether the presence of *B* *. pinnipedialis* is affecting the population dynamics of the hooded seal is unknown and increased knowledge about the strains ability to establish persistent infection and cause pathology is warranted.

The infection biology of marine mammal brucellae is to a large extent unknown, and unidentified hosts or reservoirs in the marine environment may exist. *Brucella melitensis* was isolated from Nile catfish ( *Clarias* *gariepinus* ) [16], while *B* *. ceti* and *B* *. pinnipedialis* have been isolated from lungworms in cetaceans [17] and pinnipeds [18] respectively, pointing at other possible reservoirs in the marine ecosystem besides pinnipeds and cetaceans. In addition, *Brucella* *microti* has been isolated from soil [19] and potentially novel brucellae have been isolated from frogs [20,21], indicating an ecological range of the bacteria also including cold blooded animals and possibly the environment.

*Brucella* spp. can survive and replicate within membrane-bound compartments in phagocytes and epithelial cells [22–24]. Marine mammal brucellae were previously shown to display differential responses when infecting macrophages *in vitro* [25]. Molecular studies have repeatedly characterized the hooded seal brucellae as diverging from other pinniped brucellae [6,26,27], and experimental infection of human and murine macrophage cell lines have indicated that the hooded seal strains might not have the ability to enter macrophages [25].

We infected primary alveolar macrophages from hooded seal with a *B* *. pinnipedialis* hooded seal isolate in order to investigate the infective capacity of *B* *. pinnipedialis* in a species considered to be the primary host, and by this to better understand the pathogenicity and impact of *B* *. pinnipedialis* in the hooded seal. Additionally the hooded seal macrophages were infected with *B* *. pinnipedialis* reference strain (NCTC 12890) isolated from a harbor seal ( *Phoca* *vitulina* ), *B* *. ceti* reference strain (NCTC 12891) isolated from a harbor porpoise ( *Phocoena* *phocoena* ) and a *B* *. ceti* strain isolated from an Atlantic white-sided dolphin ( *Lagenorhynchus* *acutus* ) (M83/07/1), to evaluate whether brucellae from different marine mammal species can establish an infection in hooded seal alveolar macrophages. The rationale behind including *B* *. ceti* Atlantic white-sided dolphin isolate (M83/07/1) is that this is the marine *Brucella* spp. that shows the most pathological changes in cetaceans [4].

## Materials and Methods

### Reagents and media

RPMI 1640 culture medium, Dulbecco’s minimum essential medium, Hanks’ balanced salt solution (HBSS), penicillin/streptomycin, gentamicin, Triton X-100, Histopaque 1077 and latex beads (carboxylate-modified, fluorescent red/green) were purchased from Sigma-Aldrich, St. Louis, USA. Fetal bovine serum (FBS) and Amniomax II were from Gibco, Life Technologies, Paisley, UK. Diff-Quik was from Medion Diagnostics, Düdingen, Switzerland. Sheep blood agar was from Oxoid, Oslo, Norway. Tryptic soy agar (TSA) was from Merck Millipore, Darmstadt, Germany. Mouse anti-human CD14 (Tük-4, R-PE-conjugated) monoclonal antibody (mAb) was a kind gift from Prof Victor PMG Rutten, Division of Immunology, Department of Infectious Diseases and Immunology, Faculty of Veterinary Medicine, University of Utrecht. Mouse anti-human CD14 mAb (61D3) and goat anti-mouse IgG1 antibody (R-PE-conjugated) were from Southern Biotech, Birmingham, USA. Rat anti-dog MHC class II mAb (YKIX334.2, FITC-conjugated), mouse anti-dog CD18 mAb (CA1.4E9) and mouse anti-dog CD11c mAb (CA11.6A1) were from AbD Serotec, Kidlington, UK. Rabbit polyclonal anti- *Brucella* antibody was kindly provided by Prof. JJ Letesson, Faculté Universitaire Notre Dame de la Paix, Namur, Belgium. Goat anti-mouse and goat anti-rabbit IgG1 antibodies (Alexa Fluor 488 and Alexa Fluor 546), Lysotracker Red and Live/Dead Fixable far red stain were from Molecular Probes, Life Technologies, Paisley, UK. Fluorescence mounting medium was from Dako, Glostrup, Denmark. DRAQ5 was purchased from Cell Signaling, Danvers, USA. FACS lysing buffer was from BD Biosciences, Franklin Lakes, USA. *Brucella abortus* antiserum was obtained from Remel, Europe Ltd, Kent, UK. The CytoTox 96 Non-Radioactive Cytotoxicity Assay and the Griess Reagent System were from Promega, Madison, USA.

### Hooded seal alveolar macrophages

Hooded seals from the Northeast Atlantic stock were captured as weanlings in the pack ice north-west of Jan Mayen (at about 71° 40’N, 14° 20’W) and kept in approved facilities (two 40,000 l indoor seawater pools) at the Department of Arctic and Marine Biology, University of Tromsø, Norway, for other research purposes. The capture of animals was approved by The Ministry of Foreign Affairs of Denmark, The Ministry of Education, Research and Nordic Cooperation in Nanoq (Greenland) and The Norwegian Directorate of Fisheries. Import of the captured animals was approved by The Norwegian Food Safety Authority. Although the hooded seal is classified as vulnerable in the Red List of Threatened Species, approval was given to capture a small number of animals as ongoing research involving material sampled from these animals may enlighten the sudden reduction of this species. The care of the animals was as previously described [28]. All animal use was in accordance with the Norwegian Animal Welfare Act and the regulations for use of animals in experimentation. A separate approval for sampling of tissues post mortem is not required; however biopsy sampling of live animals and method of sacrifice were approved by the National Animal Research Authority of Norway (permit no. 2402). All animals were euthanized in accordance with the Norwegian Animal Welfare Act; through stunning (given an overdose of pentobarbital (Nembutal, 20 mg kg^-1^) injected into the extradural intravertebral vein) immediately followed by bleeding. BAL was performed post mortem on a total of 9 animals euthanized at 2–30 months of age. A sterile catheter (CH 14 (53 cm), Unomed, London, UK) was placed through an insertion in the trachea. Cold, sterile PBS containing 2 mM EDTA, 20 U/ml nystatin, 100 IU/ml penicillin, 100 µg/ml streptomycin, pH 7.4, was infused in batches of 35–40 ml to a total of 450 ml/animal. The fluid was passed 2–3 times through the lungs before being retrieved and kept on ice. The BAL liquid was filtered once through sterile gauze to remove particulate matter before centrifugation at 800 x g for 10 min at room temperature (RT). The supernatant was decanted and the cell pellet washed twice in 25 ml HBSS and centrifuged at 800 x g for 10 min before the cells were counted and re-suspended in cryogenic medium (80% FBS, 10% RPMI, 10% DMSO) at 10^6^ cells/ml for storage in liquid nitrogen.

### Cell staining

BAL cell monolayers were cultivated on glass coverslips in 12 well plates (1 x 10^5^ cells/well) for 5–7 days, fixed for 15 min at RT using 4% paraformaldehyde (0.02 M sucrose, pH 7.2), washed once in PBS and stained with Diff-Quik according to the manufacturer’s instructions.

### Flow cytometry

Cell surface marker expression was assayed by flow cytometry. Phenotyping of BAL cells was carried out on ice in PBS with 0.5% BSA and 10 mM NaN_3_ (flowbuffer), using 96 well trays with U-shaped bottom. Cells were incubated with mAbs against the surface markers CD14 (Tük-4, diluted at 1:10), CD11c (diluted at 1:5), MHC class II (diluted at 1:5), and CD18 (diluted at 1:10) for 30 min. Goat anti-mouse IgG1 antibody (Alexa 488) and goat anti-mouse IgG1 antibody (RPE-conjugated), both diluted at 1:250, was added to samples containing CD11c and CD18 labeling respectively, after 3x wash in flowbuffer and blocking with 10% goat serum. Staining for live/dead cells was executed according to the manufacturer’s instructions. Cells were analysed in a FACS Calibur (BD Biosciences), using Kaluza software v.1.2 (Beckman Coulter) and gating for viable mononuclear cells in the forward scattered (FSC)/side scattered (SSC) plot. Hooded seal blood mononuclear cells (PBMCs), isolated by Histopaque 1077, were used as a positive control for cross-reaction of primary antibodies. Stained cells not examined until the next day were kept in FACS lysis buffer at 4 °C protected from light.

### Immunocytochemistry

BAL cells were seeded in eight chambered Nunc LabTek^TM^ coverglass (approximately 10,000 cells/well) or grown on glass coverslips in 12 well plates (10^5^ cells/well) and incubated at 37 °C, in an aerobic atmosphere containing 5% CO_2_ for 24 h. The cells were washed once in PBS to remove non-adherent cells and further incubated in fresh medium for 5–7 days. Adherent cells were fixed for 15 min at RT using 4% paraformaldehyde (0.02 M sucrose, pH 7.2) and washed once in PBS. Immune labeling was performed using mouse anti-human CD14 (61D3) antibody, diluted at 1:100, and mouse anti-dog CD18 antibody, diluted at 1:200. Secondary antibodies were Alexa Fluor 488 goat anti-mouse IgG and Alexa Fluor 546 goat anti-mouse IgG, diluted at 1:500. For verification of intracellular bacterial localization BAL cells were challenged with *B* *. pinnipedialis* hooded seal strain at a MOI of 50 as described in the gentamicin protection assay. Infected cells were incubated with 75 nM LysoTracker Red for 1 h prior to fixation. The cells were fixed for 15 min at RT using 4% paraformaldehyde (0.02 M sucrose, pH 7.2) at desired time points following bacterial exposure and washed once in PBS before permabilization in 0.1% Triton X-100 for 4 min. Immune labeling was done using rabbit polyclonal anti- *Brucella* spp. antibody, diluted at 1:100. Secondary antibody was Alexa Fluor 488 goat anti-rabbit IgG, diluted at 1:500. Non-specific binding of anti- *Brucella* spp. antibody was controlled for in non-infected BAL-cells. The fluorescent DNA dye DRAQ5, diluted at 1:1000, was used for visualization of nuclei. Confocal microscopy was performed using a Zeiss LSM510 META system (Carl Zeiss, Obercochen, Germany) with a 40X 1.2NA water immersion lens. Three sequential channels were recorded using the following excitation and emission parameters: Alexa 488 was excited at 488 nm and fluorescence collected through a 500–550 nm BP filter; Alexa 546 and LysoTracker Red was excited at 543 nm and fluorescence collected through a 565–615 nm BP filter; DRAQ5 was excited at 633 nm and fluorescence collected in the META detector from 644–700 nm.

### Phagocytosis

For determination of phagocytic capacity, BAL cells were seeded at approximately 10,000 cells/well in eight chambered Nunc LabTek^TM^ coverglass and incubated at 37 °C, 5% CO_2_ for 24 h. The cells were washed once in PBS to remove non-adherent cells and further incubated for 5–7 days. The medium was then replaced with fresh medium containing 0.025% v/v 0.5 µm red or 0.1% v/v 2 µm green fluorescent, carboxylate-modified polystyrene, latex beads and the cells incubated for 10, 30, 60, and 180 min at 37 °C, 5% CO_2_. The cells were fixed for 15 min at RT using 4% paraformaldehyde (0.02 M sucrose, pH 7.2), washed repeatedly to remove excess beads and kept in PBS. The fluorescent DNA dye DRAQ5, diluted at 1:1000, was used for visualization of nuclei. Confocal microscopy was performed as described in paragraph *Immunocytochemistry*. For the live cell images, cells were seeded in 35 mm Ibidi µ-dishes at 10^6^ cells/dish and incubated at 37 °C, 5% CO_2_ for 7 days. After initial calibration, the medium was replaced with fresh medium containing 0.0125% v/v 0.5 µm red or 0.05% v/v 2 µm green latex beads. Pictures were obtained in a Nikon Bio Station IMq every third min for the first 3 h, every fifth min for another hour, and every tenth min for 2 h for a total of 6 h.

### Bacterial strains and growth conditions

The *Brucella* strains used were a *B* *. pinnipedialis* hooded seal isolate; animal number 17, spleen [14], from now on entitled B17, *B* *. pinnipedialis* reference strain (NCTC 12890) isolated from a harbor seal, *B* *. ceti* reference strain (NCTC 12891) isolated from a harbor porpoise, a *B* *. ceti* strain M83/07/1 isolated from an Atlantic white-sided dolphin (all from G. Foster, Scottish Agricultural College, Consulting Veterinary Services, Inverness, UK) and *B. suis* 1330 (NCTC 10316) (provided by B. Djønne, Norwegian Veterinary Institute, Oslo, Norway). The strains were kept at -80 °C on Microbank™ beads (Pro-Lab Diagnostics, Round Rock, USA). Before each assay a bead was taken from one specific Microbank™ batch and plated on sheep blood agar at 37 °C in a 5% CO_2_ atmosphere for 2–4 days, the strains were thereafter plated again on sheep blood agar and grown at 37 °C in a 5% CO_2_ atmosphere for 48 h for *B. suis* 1330 and 96 h for the marine strains (serial dilutions of the bacteria in sterile PBS showed that the strains had reached the log phase; 10^9^ bacteria/ml when OD 600 nm = 1). We verified the expression of smooth surface antigens for the Microbank™ batches by visual inspection of colony morphology, crystal violet staining and agglutination with antiserum to smooth *B. abortus* [29,30]. An inoculum yielding an approximate multiplicity of infection (MOI) of 50 was prepared by diluting bacteria in PBS to an OD of 1 and thereafter adding the correct amount from this solution to RPMI supplemented with 10% FBS. The identity of the strains was verified by PCR and gel electrophoresis with marine mammal brucellae specific primers designed to amplify the *bp26* gene [31] and the primer sets Bruce 11, 18 and 45 from the MLVA-15 assay [32,33]. The sensitivity towards gentamicin in the marine mammal *Brucella* spp. was compared to *B. suis* 1330 (Figure S1).

### Gentamicin protection assay

BAL cells were seeded (1.25 x 10^5^ cells/well) in 24 well plates and cultured in Amniomax II. After 24 h the medium was changed and the cells washed once with PBS to remove non-adherent cells. The gentamicin protection assay was initiated after approximately 48–72 h, when the cells started to appear fully adherent (flattened, cytoplasma spreading). The cells were challenged with *Brucella* spp. at a MOI of 50 for 1 h and incubated at 37 °C supplemented with 5% CO_2_. As Amniomax II contains gentamicin, the bacteria were suspended in RPMI supplemented with 10% FBS. The infection was synchronized by centrifugation at 230 x g for 10 min in RT, and ended by rinsing the wells twice with PBS and refilling with 1 ml of Aminomax II containing 100 µg/ml gentamicin to kill extracellular bacteria. After 1 h the medium was replaced with Amniomax II containing 35 µg/ml gentamicin, in which the cells were incubated for specified times (1.5, 7, 24, 48 h). Before harvesting of intracellular bacteria, the cells were washed three times with PBS to remove remaining antibiotics. Macrophage cell membranes were disrupted using 300 µl/well of 0.1% Triton X-100 in PBS followed by incubation at 37 °C for 10 min. Sterile mini cell scrapers were used to ensure complete detachment of cells (Leap Biosciences, Palo Alto, USA) and the lysate was repeatedly pipetted to aid macrophage membrane disruption. Blood agar plates were inoculated in duplicate with 100 µl each of lysate in serial dilutions and evaluated for the presence of colony forming units (CFU). Supernatants were also plated to control the efficiency of extracellular bacterial killing by gentamicin. The influence of gentamicin on intracellular infection dynamics of *B* *. pinnipedialis* reference strain (NCTC 12890) and hooded seal isolate (B17) was determined (Figure S2). Potential toxic cell damage was measured by quantitatively determining the release of lactate dehydrogenase using the CytoTox 96 Non-Radioactive Cytotoxicity Assay and production of NO following infection was assessed using the Griess Reagent System according to the manufacturer’s instructions. Absorbance was read using an Epoch Microplate Spectrophotometer (BioTek, Winooski, USA).

### Statistical analysis

Group data were compared using Student’s *t* test for independent samples. Differences were considered significant for *p* values of < 0.05.

## Results

### Cell morphology

**Figure 1:**
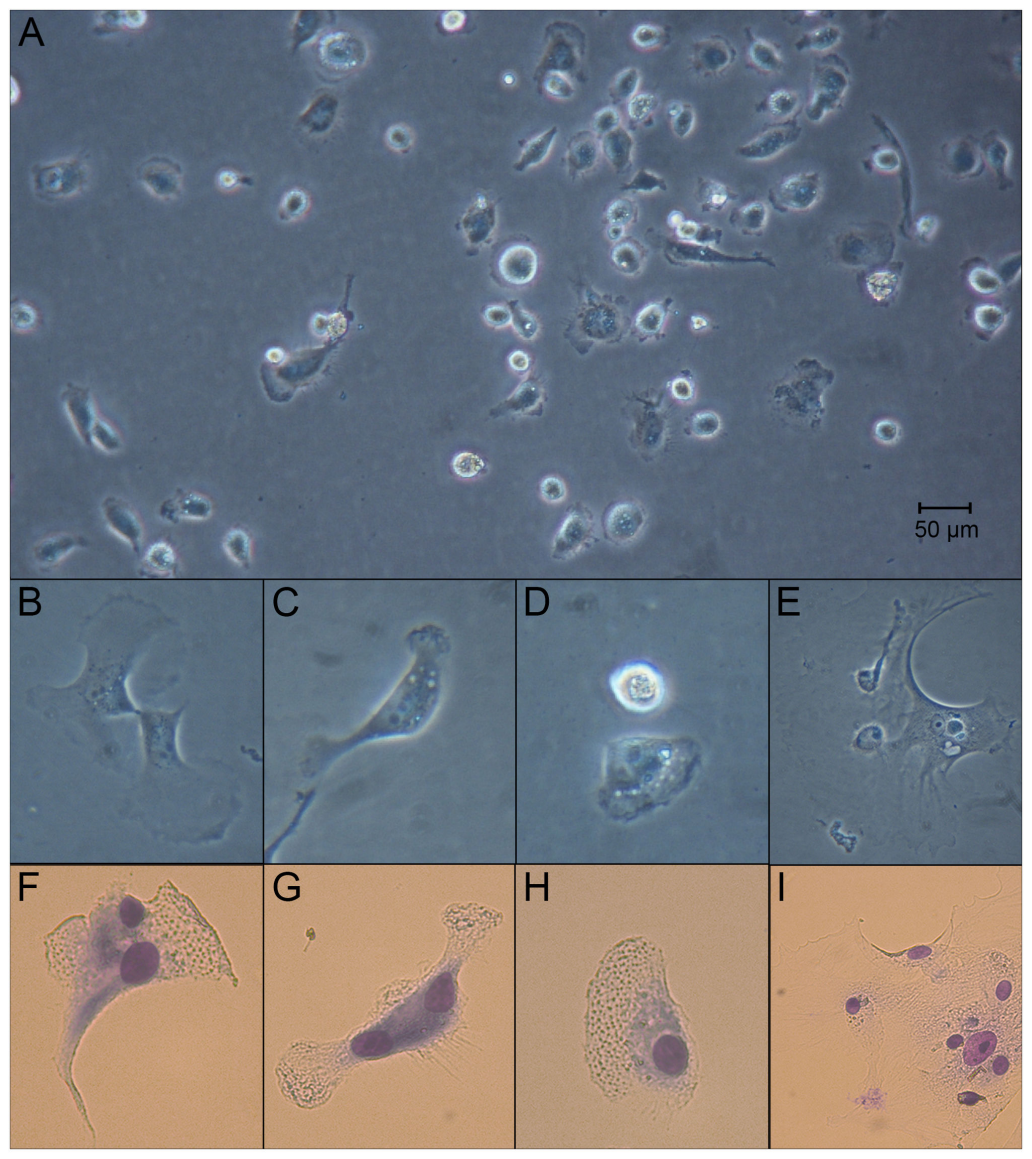
Cell morphology. Morphology of hooded seal BAL cells cultured for 5–7 days in Amniomax II. Adherent cells are rounded and reflect light 24 h after initiation of the culture. A macrophage-like pleomorphic appearance with many cells displaying a fan- or veil shaped cytoplasm with an irregular, ruffled border emerges after 2–3 days (A). Vacuoles are present in the cytoplasm, often near the nucleus and the nucleoli are distinct (B–D). A–E: live cells from inverted light microscope, F–I: Diff-Quik stained cells presenting purple nuclei, light blue cytoplasm and a granular hyaloplasm (BAL; bronchoalveolar lavage).

Adherent BAL cells appeared as rounded and bright 24 h after initial seeding. Cytoplasmic protrusions started to appear after 24–48 h of culture, yielding a heterogenic picture with fan-shaped, veil-shaped, elongated and round cells (Figure 1). Cytoplasmic clear vacuoles were detected in many cells (Figure 1, C and D). Adherent BAL cells stained with Diff-Quik displayed a macrophage-like morphology with purple oval to fusiform nuclei with one or two distinct nucleoli. The cytoplasm stained light blue in the central portion of the cell and changed to clear veils of granular hyaloplasm with ruffled edges spreading about the cell (Figure 1, F–I). Live cell imaging revealed that the adherent BAL cells were motile and changed their morphological appearance by protrusion of both fan-shaped and slender, spiky pseudopods (Videos S1 and S2). During a 6 h period many cells were seen to migrate a distance of more than twice their own size.

### Macrophage identification

**Figure 2:**
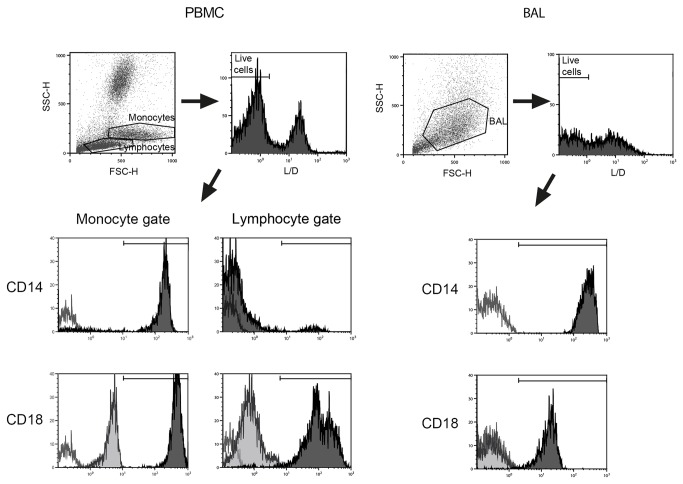
Expression of membrane markers. The expression of CD14 and CD18 on hooded seal PBMC and BAL cells by flow cytometric analysis. PBMCs (left) were divided into monocyte and lymphocyte gates, while BAL cells (right) were broadly gated to encompass all cells of likely macrophage appearance. Only cells classified as viable (using Live/Dead fixable far red stain) were routed to analysis. The respective cell populations were analyzed for CD14 and CD18 (dark grey) or unstained cells (open histograms). For CD18 an indirect staining technique was used, and secondary antibodies alone are depicted in light grey histograms; the resulting dimly positive staining were assumed to be due to unspecific binding, possibly due to Fc receptor cross-reactivity (CD14; cluster of differentiation 14, CD18; cluster of differentiation 18, PBMC; peripheral blood mononuclear cell).

**Figure 3:**
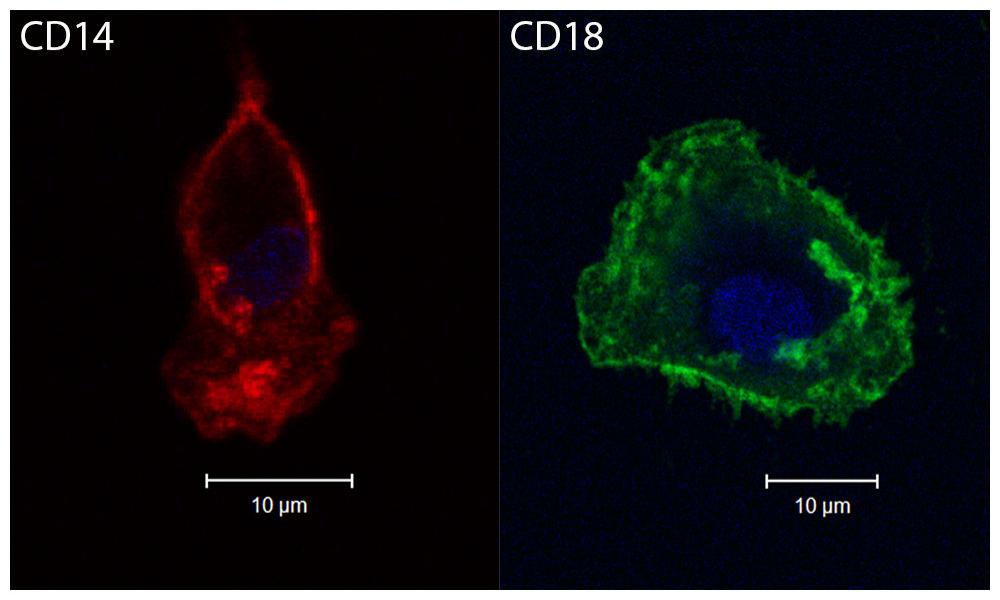
Macrophage identification. Adherent BAL cells cultured for 5–7 days were examined for the expression of membrane markers by the use of mAbs. Representative confocal micrographs showing BAL cells immunostained for CD14 (red) and CD18 (green). Nuclei are visualized by DRAQ5 (blue). Pictures are taken of wet samples using a 40X 1.2NA water immersion lens (mAbs; monoclonal antibodies).

Hooded seal BAL cells were screened for expression of surface markers typically expressed by monocytes/macrophages using flow cytometry. Since antibody reactivity to seal leukocytes has not been well studied, we assessed cross-reactivity of mAbs in more well-defined cell populations by staining PBMCs from the same animals. As expected, anti-human CD14 mAb positively stained the great majority of blood monocytes, but to a very little extent lymphocytes, as defined by monocyte and lymphocyte gating in the scatter plot (Figure 2). Also as expected, the pan-leukocyte marker CD18 was positive in both of these leukocyte fractions using a mAb raised against dog. However, anti-dog MHC II and anti-dog CD11c did not stain any of the PBMC fractions (not shown), indicating no cross-reactivity to seal. We found that virtually all BAL cells expressed both CD14 and CD18, indicative of leukocytes of myeloid origin. The flow cytometry results showed good correlation between the different animals sampled, both with respect to the BAL cells (*n* = 3) as well as the PBMCs used as control (*n* = 2). Immunocytochemistry showed that adherent BAL cells cultured for 5–7 days were positive for CD14 and CD18 (Figure 3).

### Phagocytic activity

**Figure 4:**
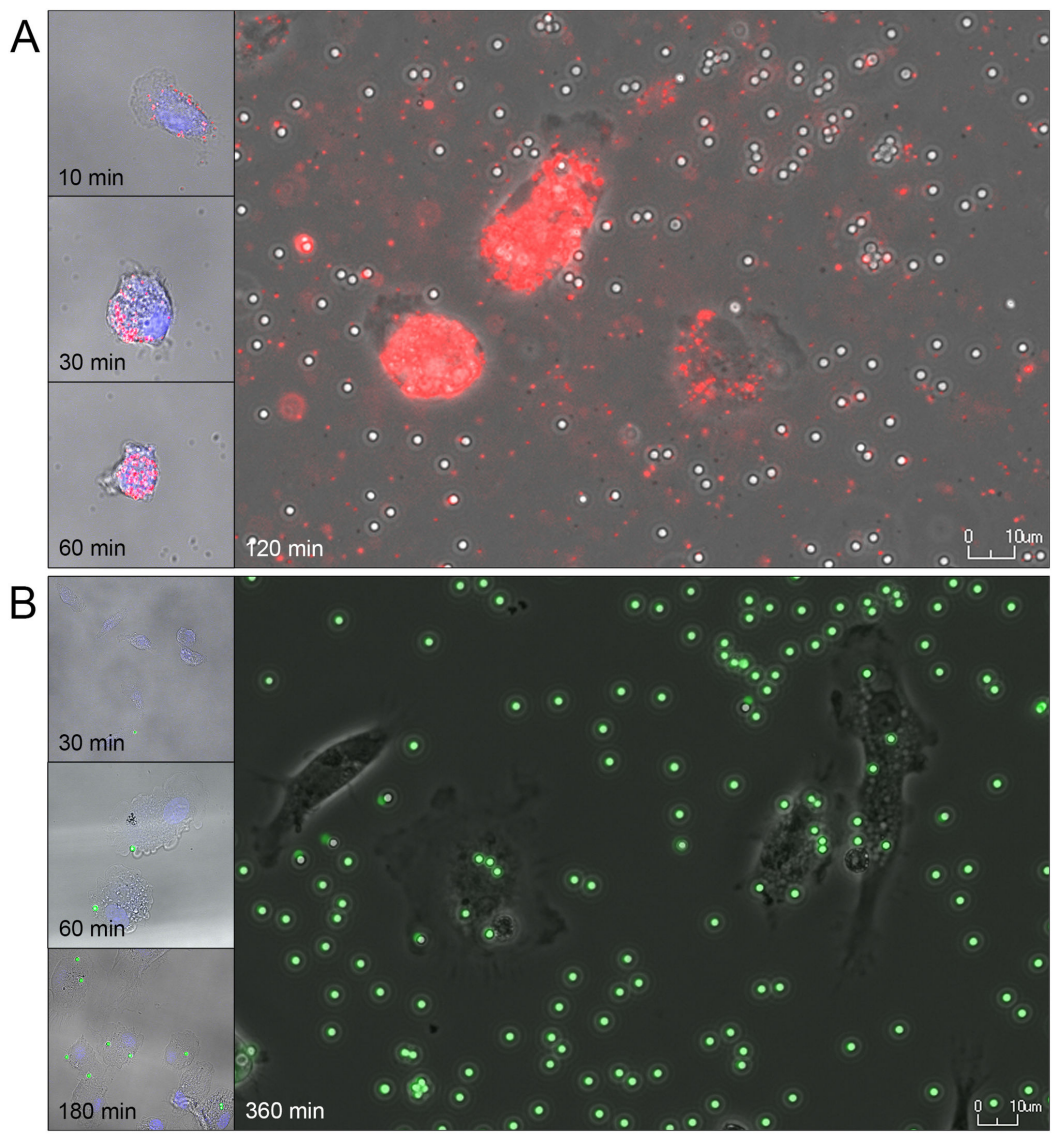
Phagocytic activity. Ingestion of fluorescent carboxylated latex beads were confirmed by confocal microscopy and live cell imaging of adherent BAL cells incubated with 0.5 µm beads (A) and 2 µm beads (B) at various time points, as indicated. After fixation the cells were labeled with DRAQ5 (blue) for visualization of the nuclei (left side, A and B). Pictures are taken of wet samples using a 40X 1.2NA water immersion lens. Live cell imaging was performed in a Nikon Bio Station IMq (right side, A and B).

Adherent hooded seal BAL cells were tested for phagocytic activity by incubation with fluorescent latex beads. Already at 10 min, BAL cells had ingested 0.5 µm latex beads (Figure 4A). The amount of internalized 0.5 µm beads increased rapidly and by 2 h of incubation the cells were completely filled. Phagocytosis of the 2 µm latex beads started later than observed for the smaller beads and was visualized from 1.5–6 h of incubation with live cell imaging (Figure 4B video S3). The amount of beads ingested as well as the total number of phagocytic cells increased with prolonged incubation. The maximum number of 2 µm beads ingested was visually counted to be six during the 6 h period of imaging (*n* = total of 60 cells from 10 different positions of imaging). A smaller fraction of the adherent BAL cells phagocytized 2 µm beads compared to the 0.5 µm beads that could be detected in nearly every cell.

### Marine mammal brucellae enter hooded seal alveolar macrophages but do not multiply

**Figure 5:**
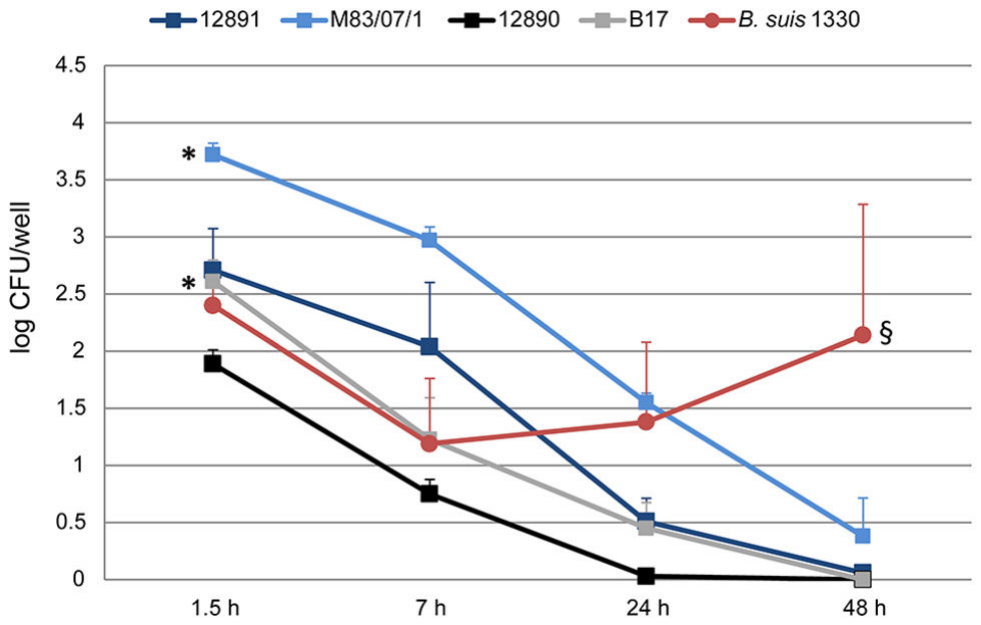
Infection dynamics of *Brucella* spp. in hooded seal alveolar macrophages. Hooded seal alveolar macrophages were challenged with *B* *. pinnipedialis* hooded seal isolate (B17), *B* *. pinnipedialis* reference strain (12890; harbor seal), *B* *. ceti* reference strain (12891; harbor porpoise)*,* *B* *. ceti* Atlantic white-sided dolphin isolate (M83/07/1) and *B. suis* 1330 at a MOI of 50 in a gentamicin protection assay. None of the marine mammal strains multiplied and all were eliminated by 48 h pi. Although the Atlantic white-sided dolphin isolate (M83/07/1) entered the macrophages in slightly higher numbers, no major differences were observed in the intracellular bacterial dynamics between the strains. *Brucella suis* 1330 presented a different pattern with an initial drop at 7 h pi followed by multiplication. Each indicator represents the mean of six (12890, B17, M83/07/1) or three (12891, *B. suis* 1330) parallels. Error bars correspond to the standard error. * (different from respective reference strain, *p* < 0.05), § (different from 12890 and B17, *p* < 0.05) (MOI; multiplicity of infection).

**Figure 6:**
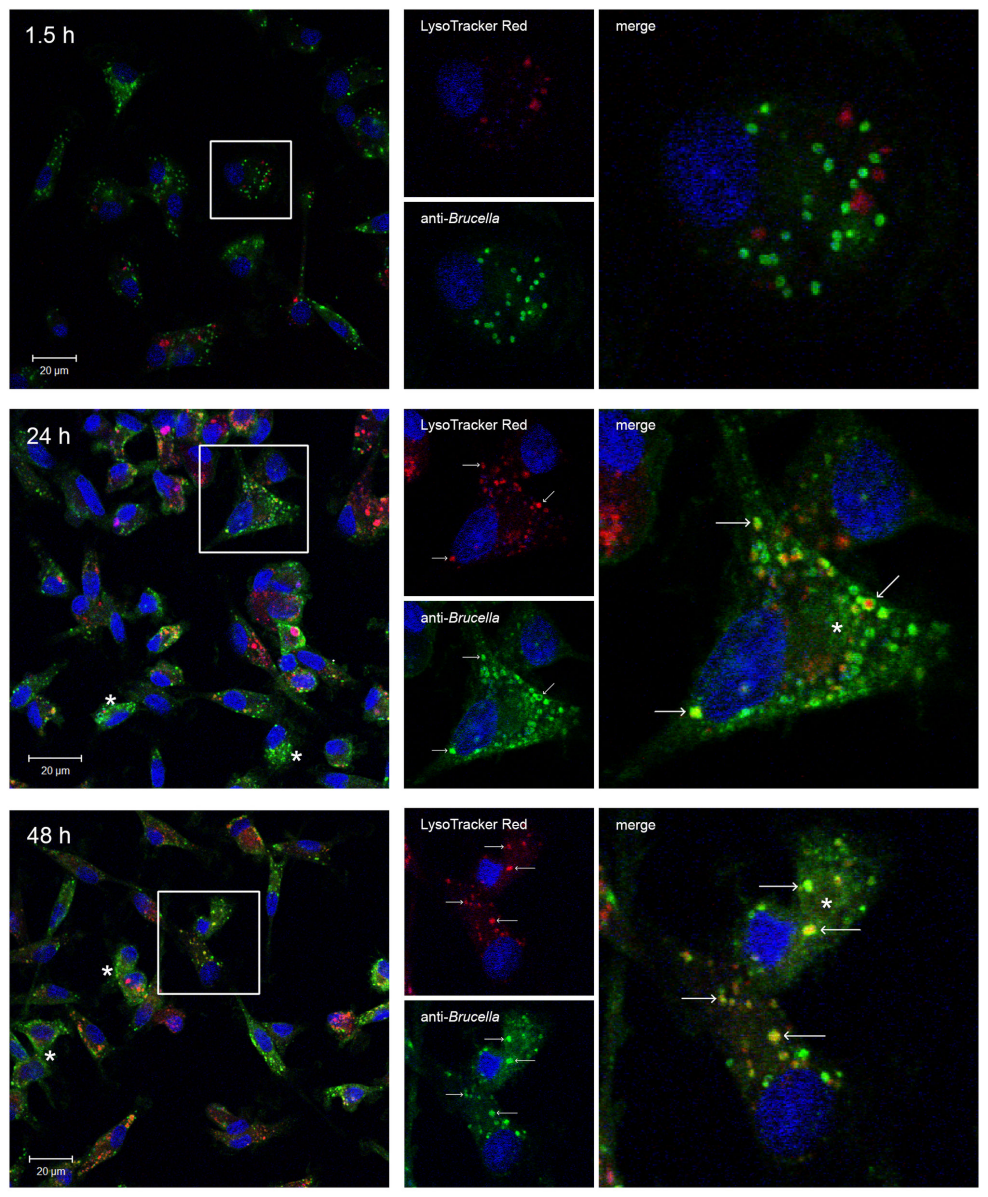
Intracellular B17 in hooded seal alveolar macrophages. Hooded seal alveolar macrophages were cultured in 12 well plates containing glass coverslips and challenged with *B* *. pinnipedialis* hooded seal isolate (B17) as described in the gentamicin protection assay. Cells were incubated with LysoTracker Red (red) for 1 h before fixed at 1.5, 24 and 48 h after exposure and immune labeled with anti- *Brucella* antibody 1:100 (green). DRAQ5 was used for visualization of the nuclei (blue). B17 are detected in nearly every cell with multiple bacteria/cell. Confocal microscopy revealed colocalization (arrows) of B17 and lysosomal compartments at 24 and 48 h. Bacterial debris scattered throughout the cytoplasm can be observed at 24 and 48 h pi (asterisk).

The results from the gentamicin protection assay revealed that all marine mammal *Brucella* strains tested were able to enter hooded seal alveolar macrophages *in vitro*. When challenging the cells with a MOI of 50, *B* *. pinnipedialis* hooded seal isolate (B17) showed a moderate ability to enter, yielding a log CFU of 2.61 at 1.5 h post infection (pi), compared to *B* *. pinnipedialis* reference strain (NCTC 12890) at a log CFU of 1.74 and *B* *. ceti* Atlantic white-sided dolphin isolate (M83/07/1) at a log CFU of 3.72 (Figure 5). Both *B* *. pinnipedialis* strains tested were eliminated rapidly and by 7 h pi the retrieved CFUs were more than halved compared to the initial numbers. By 48 h pi, both strains were completely eliminated. *Brucella* *ceti* reference strain (NCTC 12891) displayed the same pattern as the two *B* *. pinnipedialis* strains. The *B* *. ceti* Atlantic white-sided dolphin isolate showed a slightly more protruded pattern of reduction, but was almost eliminated by 48 h pi. Of six parallels in two separate assays, one well showed a slightly higher number of M83/07/1 resulting in a larger standard deviation for the 48 h time point compared to the rest. Although all four marine mammal strains were able to reside for some time intracellularly, none presented the ability to multiply. *Brucella suis* 1330 followed a different pattern compared to the marine mammal brucellae. Of total 3 wells, multiplication occurred in two wells while in one well the bacteria were eliminated. The intracellular localization of B17 was confirmed using confocal microscopy (Figure 6). Double immune labeling with a rabbit anti- *Brucella* antibody and Lysotracker Red revealed colocalization of intracellular bacteria with lysosomes at both 24 and 48 h pi. Non-specific binding of anti- *Brucella* spp. antibody was not detected (Figure S3). No release of lactate dehydrogenase was detected following infection with *B* *. ceti* reference strain or *B. suis* 1330, suggesting minimal cytotoxicity induced by this procedure. Measurement of NO was not found to be applicable for the hooded seal alveolar macrophages as stimulation with LPS (1 µg/ml for 1.5, 7, 24, and 48 h) failed to elicit detectable nitrite production.

## Discussion

For the first time, we present the results of infecting hooded seal alveolar macrophages with marine mammal *Brucella* spp. *in* *vitro*. Cells retrieved by BAL in hooded seals were found to be positive for the monocyte/macrophage membrane marker CD14 [34], as well as the pan-leukocytic marker CD18 [35]. Adherent cells cultured from BAL samples displayed a macrophage-like morphology, were CD14 and CD18 positive, and performed phagocytosis of latex beads. Based on these characteristics, we concluded that adherent cells cultured from hooded seal BAL samples were primary alveolar macrophages.

Studies of the mechanisms of bacterial invasion and intracellular multiplication involving the marine mammal brucellae are scarse, and existing information are collected from human and murine macrophage cell lines [25]. According to the results from these *in vitro* assays, the *B* *. pinnipedialis* hooded seal strain seems to have a restricted, if any, ability to establish chronicity as the bacteria fail to multiply intracellularly. *Brucella* *pinnipedialis* isolated from harbor seal has shown variation in the ability to multiply in human and murine macrophages. The reference strain (NCTC 12890) multiplied with a pattern similar to pathogenic terrestrial brucellae, while another harbor seal isolate (M2533) was eliminated by 48 h pi in the work by Maquart and co-workers [25]. Recently, *B* *. pinnipedialis* hooded seal strain as well as the reference strain (NCTC 12890) was shown to be almost completely eliminated by 72 h pi using the same cell lines (Larsen and Nymo, unpublished results). Little information is available regarding the pathogenicity of these bacterial strains in seals, which are assumed to be the natural hosts of *B* *. pinnipedialis* , and the ability of the marine mammal brucellae to enter and multiply in host cells has been unexplored. This led us to investigate if the lack of intracellular multiplication may be due to host specific characters absent in the human and murine macrophage cell lines but present in hooded seal macrophages. To this end we isolated hooded seal alveolar macrophages and challenged them with a *B* *. pinnipedialis* hooded seal isolate (B17). To evaluate possible host specificity in the marine mammal brucellae, the hooded seal alveolar macrophages were also challenged with *B* *. pinnipedialis* reference strain (NCTC 12890), originally isolated from a harbor seal, *B* *. ceti* reference strain (NCTC 12891), originally isolated from a harbor porpoise and a *B* *. ceti* Atlantic white-sided dolphin isolate (M83/07/1). The results from the gentamicin protection assay revealed that all four marine mammal brucellae strains were able to enter hooded seal alveolar macrophages in culture. Both *B* *. pinnipedialis* strains and the *B* *. ceti* reference strain entered the cells in lower numbers compared to the *B* *. ceti* Atlantic white-sided dolphin isolate. Group data analysis showed that at 1.5 h pi B17 and M83/07/1 are significantly different from their respective reference strains; however the amount of intracellular bacteria at the start of the infection does not influence on the outcome of infection. Contrary to what could be expected, none of the strains were able to multiply intracellularly and were completely eliminated by 48 h pi. As hooded seal are believed to be the primary host of *B* *. pinnipedialis* hooded seal strain, it is intriguing that the hooded seal isolate were not able to multiply. Colocalization of B17 with lysosomal departments was observed at both 24 and 48 h pi. Additionally, at the same time points, bacterial debris appears scattered throughout the infected cells indicating bacterial degradation, as previously reported for an attenuated *B. abortus* strain [23,36]. This is line with the observed reduction of viable intracellular bacteria retrieved at the later stages after infection.

Extracellular gentamicin has been shown to affect the fate of intracellular *Brucella* spp. [37] as well as other intracellular bacteria [38,39]. To control for a possible intracellular bactericide effect of gentamicin, we kept murine macrophages infected with *B* *. pinnipedialis* reference strain and hooded seal isolate in medium with different concentrations of gentamicin. The outcome of infection was not altered, and none of the strains multiplied within 48 h pi, also not when gentamicin was completely removed (Figure S2). Compared to *B. suis* 1330, the marine mammal *Brucella* spp. have a reduced sensitivity towards gentamicin (Figure S1). *Brucella suis* 1330 as well as *B. abortus* are previously shown to multiply intracellularly in assays using substantial amounts of gentamicin [40,41]. Hence, extracellular gentamicin in the concentration implemented herein does not seem to affect the intracellular fate of marine mammal *Brucella* spp.

In contrast to their parental smooth bacterial strains, engineered rough mutants are eliminated after cellular entry. This is believed to be due to macrophage activation resulting in bacterial eradication [42,43] as well as bacterial destruction following cellular necrosis or oncosis [44,45]. The marine mammal *Brucella* spp. express a smooth-type lipopolysaccharide [46], although there are some indications that at least *B* *. ceti* may appear as a rough phenotype [5,47]. To exclude that intracellular elimination could be attributable to transition from smooth to rough phenotype, all strains used were confirmed to express a smooth morphology both before and after the gentamicin protection assay was performed. As no apparent morphological changes indicating necrosis or cell death were observed in the infected cells and release of lactate dehydrogenase was not detected, the reduced number of bacteria retrieved is very likely due to intracellular eradication and lack of concomitant multiplication.

Production of nitric oxide (NO) is often measured as an indicator of cellular activation during infection [48]. Stimulation of hooded seal alveolar macrophages with LPS or bacterial challenge did not elicit measurable levels of nitrite (indicator of NO production) and could not be applied to evaluate cellular activation following infection with marine *Brucella* spp. Our finding is in line with previous reports regarding human macrophages [49]. However; as nitric oxide is detected in canine macrophages [50] and recently found to mediate differential killing of *Mycobacterium bovis* in bovine macrophages [51], this contrasting discovery is interesting and important to include when evaluating immune mechanisms in intracellular bacterial eradication of marine mammal brucellae in host cells.

Although interaction with different cell types are shown for the pathogenic terrestrial *Brucella* spp., macrophages are believed to be preferred as long time survival in the mononuclear phagocyte system of spleen, liver and bone marrow will sustain a chronic infection [52,53]. In the work of Tryland et al. [14], *B* *. pinnipedialis* hooded seal strain was isolated from spleen and lung lymph nodes, tissues known to be rich in macrophages, but intracellular presence was not investigated. Although isolation from multiple tissues could be suggestive of a bacteremia, pathological changes due to *Brucella* -infection have to date not been reported in hooded seals. It seems intriguing that *B* *. pinnipedialis* could be recovered from multiple organs throughout the body without intracellular replication, but during an acute stage of the infection this could be possible.

Another potential explanation is that *B* *. pinnipedialis* is multiplying in different host cells besides macrophages. Preliminary results indicate that *B* *. pinnipedialis* hooded seal strain is quickly eliminated from infected hooded seal PBMCs, reaching lysosomal compartments already at 1 h pi (Larsen, unpublished results). Multiplication could take place in epithelial cells, as shown for terrestrial pathogenic brucellae [23], with macrophages picking up the bacteria and clearing a possible acute infection after bacterial release from host cells. This last scenario could indicate that *B* *. pinnipedialis* might not cause chronic infections in seals due to lack of survival in mononuclear phagocytic cells. Provided that *B* *. pinnipedialis* is unable to multiply intracellularly in any cells derived from seal, it could be argued that seals may not be the primary host for *B* *. pinnipedialis* , but rather a “dead-end” or spillover host being susceptible to infection derived from other hosts in the marine environment.

*Brucella* *ceti* has been detected in connection with numerous pathological findings in cetaceans [5]. When isolated from Atlantic white-sided dolphin, *B* *. ceti* is commonly retrieved from multiple organs [4], indicating that the bacteria in this particular host has a potential to disseminate throughout the body. Whether this is due to properties of the strain or the host is unknown. The fact that both *B* *. ceti* strains were completely eliminated shortly after entry in the hooded seal alveolar macrophages could reflect host specificity and further studies involving cetacean macrophages needs to be performed in order to draw firm conclusions. A strong host preference for the marine brucellae in their natural environment could suggest that exposure to *Brucella* spp. may be important for marine mammals, but that persistent infection is restricted to preferential hosts.

In conclusion, this work shows that two different *B* *. pinnipedialis* strains and two *B* *. ceti* strains were able to enter primary hooded seal alveolar macrophages *in vitro*. None of the strains multiplied intracellularly and all were eliminated by 48 h pi. The fact that *B* *. pinnipedialis* reference strain and both *B* *. ceti* strains were eliminated shortly after entry into the hooded seal alveolar macrophages could reflect host specificity rather than lack of ability to multiply intracellularly in preferential host specific macrophages. Although the infection biology *in vivo* is still not clear, these data increase our understanding of the pathogenicity of *B* *. pinnipedialis* in hooded seal. The hooded seal strain has never been isolated from any other marine mammal species, suggesting a strong host specificity or host/pathogen association. Macrophages are known to be the replicative niche of *Brucella* spp. and support the chronicity of *Brucella* infection, which is the hallmark of brucellosis. However, our results suggest that the *B* *. pinnipedialis* hooded seal strain is not able to multiply and induce a chronic infection in hooded seal macrophages. Therefore, the hooded seal may not be a reservoir species but rather a spillover host, suggesting that these *Brucella* strains exists in a niche in the environment, to which hooded seals, and not other seal species, are exposed. Infection assays involving other cell types from the hooded seal and other seal species, as well as investigation of intracellular trafficking are warranted in order to gain more information on the marine mammal brucellae intracellular lifestyle and to identify true reservoir hosts.

## Supporting Information

Video S1 Live images of hooded seal alveolar macrophages *in vitro*. BAL cells were cultured in Ibidi µ-dishes for 7 days prior to the experiment. After initial calibration in a Nikon Bio Station, the cells were incubated with medium containing 2 µm green fluorescent latex beads (0.05% v/v). Pictures were obtained as stated in M & M and the cells were observed for a total of 6 h. In video 1 an area of 10 cells is included using 20X magnification. Cell migration as well as protrusion of both fan-shaped and slender, spiky pseudopods are visualized.
(MP4)

Video S2 Live images of hooded seal alveolar macrophages *in vitro*. Video 2 is a subset of the area filmed in video 1, showing 7 of the same cells at 40X magnification.
(MP4)

Video S3 Live images of phagocytizing hooded seal alveolar macrophages *in vitro*. Live images of cultured BAL cells were obtained as described for Videos S1 and S2. Five BAL cells can be observed performing phagocytosis of 2 µm green fluorescent latex beads. A maximum of six beads were ingested during a period of 6 h (cell number two counting from the left of the screen). Ingestion of dead cell debris are also observed (first cell counting from the left; round 10-15 and cell number two counting from the left of the screen; round 80–85).
(MP4)

Figure S1 Gentamicin sensitivity. *B. suis* 1330, *B* *. pinnipedialis* reference strain (12890), *B* *. pinnipedialis* hooded seal isolate (B17), and *B* *. ceti* Atlantic white-sided dolphin isolate (M83/07/1) were tested for differences in sensitivity towards gentamicin. 10^7^ bacteria diluted in MEM with 10% FBS were incubated with different concentrations of gentamicin (100, 50, 25, 12.5, 6.25, and 3.125 µg/ml) for 1 h at 37°C, 5% CO_2_. The bacterial inoculum was prepared as described in M & M section *Bacterial strains and growth conditions*. The amount of viable bacteria post incubation was determined by plating the bacterial suspension in serial dilutions on tryptic soy agar and evaluated for the presence of colony forming units (CFU). Each concentration was tested in duplicates. Results are depicted in present remaining viable bacteria. Error bars correspond to the standard error.
(TIF)

Figure S2 Influence of gentamicin on intracellular infection dynamics of *B* *. pinnipedialis* . Murine macrophages (RAW264.7; ATCC no. TIB-71) were challenged with *B* *. pinnipedialis* reference strain (12890) and *B* *. pinnipedialis* hooded seal strain (B17) at a MOI of 50 in a gentamicin protection assay as described in M and M. Following incubation with 100 µg/ml gentamicin for 1 h to kill extracellular bacteria, the cells were incubated with different concentrations of gentamicin (0, 10, and 35 µg/ml) for 48 h pi. Although intracellular B17 seems to be eliminated at a slightly lower speed when incubated in medium without gentamicin, none of the strains multiplied by 48 h pi. No release of lactate dehydrogenase was detected following infection and gentamicin-treatment of the cells, suggesting minimal cytotoxicity induced by these procedures. Each indicator represents the mean of three parallel wells. Error bars correspond to the standard error (MOI; multiplicity of infection).
(TIF)

Figure S3 Anti-*Brucella* negative control. Hooded seal alveolar macrophages were cultured in 12 well plates containing glass coverslips for 5–7 days. Cells were incubated with LysoTracker Red (red) for 1 h before fixed and immune labeled with anti-*Brucella* antibody 1:100 (green). DRAQ5 was used for visualization of the nuclei (blue). Confocal microscopy revealed no unspecific binding of anti-*Brucella* antibody in non-infected cells.
(TIF)
