# Supplementary figures and images for: Entry and Elimination of Marine Mammal Brucella spp. by Hooded Seal (Cystophora cristata) Alveolar Macrophages In Vitro
Source: PLoS One. 2013 Jul 25;8(7):e70186. doi: 10.1371/journal.pone.0070186 (PMC3723690; doi:10.1371/journal.pone.0070186)

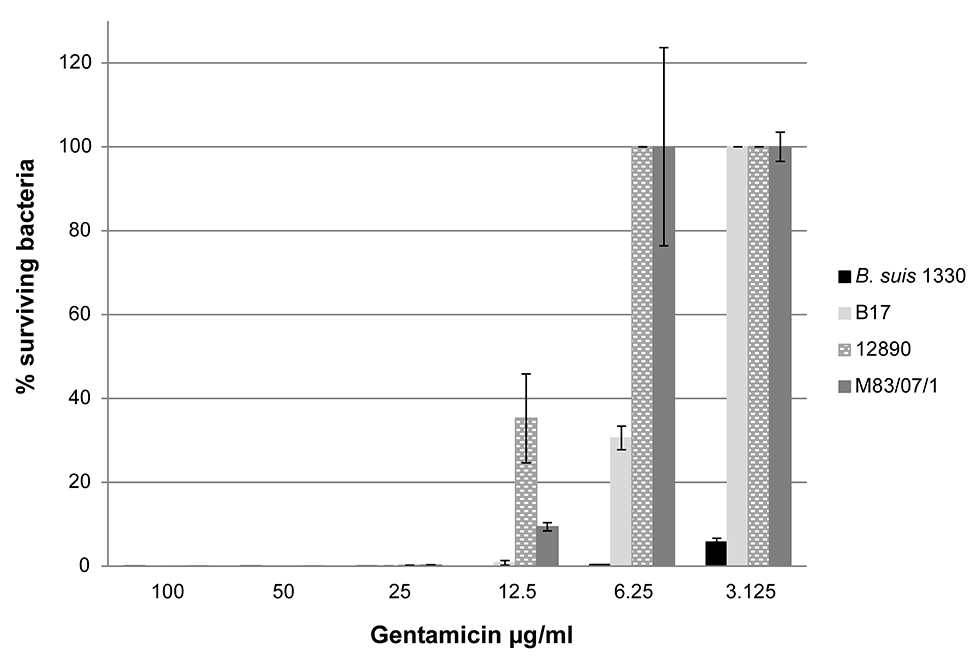

Supplement: Figure S1 — B. suis 1330, B . pinnipedialis reference strain (12890), B . pinnipedialis hooded seal isolate (B17), and B . ceti Atlantic white-sided dolphin isolate (M83/07/1) were tested for differences in sensitivity towards gentamicin. 107 bacteria diluted in MEM with 10% FBS were incubated with different concentrations of gentamicin (100, 50, 25, 12.5, 6.25, and 3.125 µg/ml) for 1 h at 37°C, 5% CO2. The bacterial inoculum was prepared as described in M & M section Bacterial strains and growth conditions. The amount of viable bacteria post incubation was determined by plating the bacterial suspension in serial dilutions on tryptic soy agar and evaluated for the presence of colony forming units (CFU). Each concentration was tested in duplicates. Results are depicted in present remaining viable bacteria. Error bars correspond to the standard error. (TIF) [file pone.0070186.s004.tif]

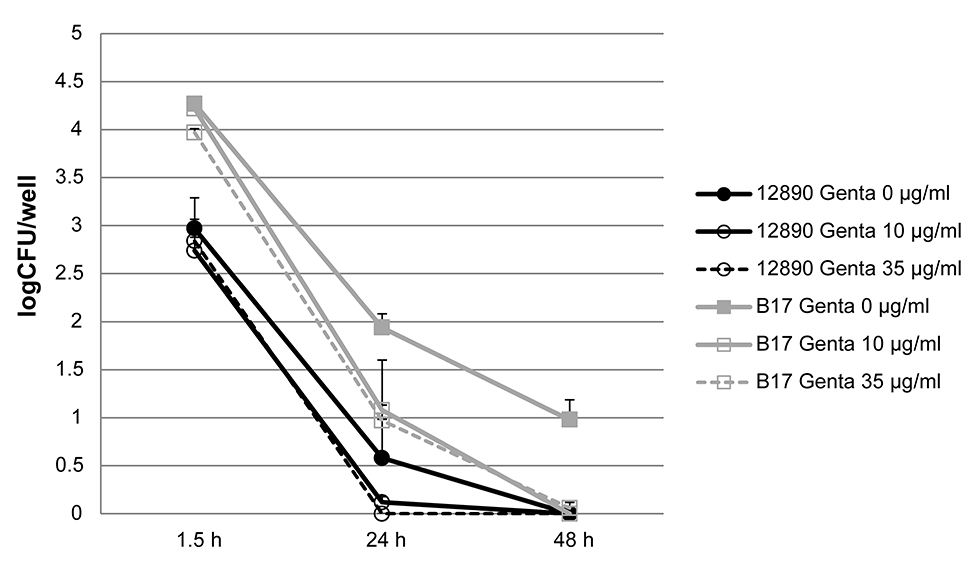

Supplement: Figure S2 — Murine macrophages (RAW264.7; ATCC no. TIB-71) were challenged with B . pinnipedialis reference strain (12890) and B . pinnipedialis hooded seal strain (B17) at a MOI of 50 in a gentamicin protection assay as described in M and M. Following incubation with 100 µg/ml gentamicin for 1 h to kill extracellular bacteria, the cells were incubated with different concentrations of gentamicin (0, 10, and 35 µg/ml) for 48 h pi. Although intracellular B17 seems to be eliminated at a slightly lower speed when incubated in medium without gentamicin, none of the strains multiplied by 48 h pi. No release of lactate dehydrogenase was detected following infection and gentamicin-treatment of the cells, suggesting minimal cytotoxicity induced by these procedures. Each indicator represents the mean of three parallel wells. Error bars correspond to the standard error (MOI; multiplicity of infection). (TIF) [file pone.0070186.s005.tif]

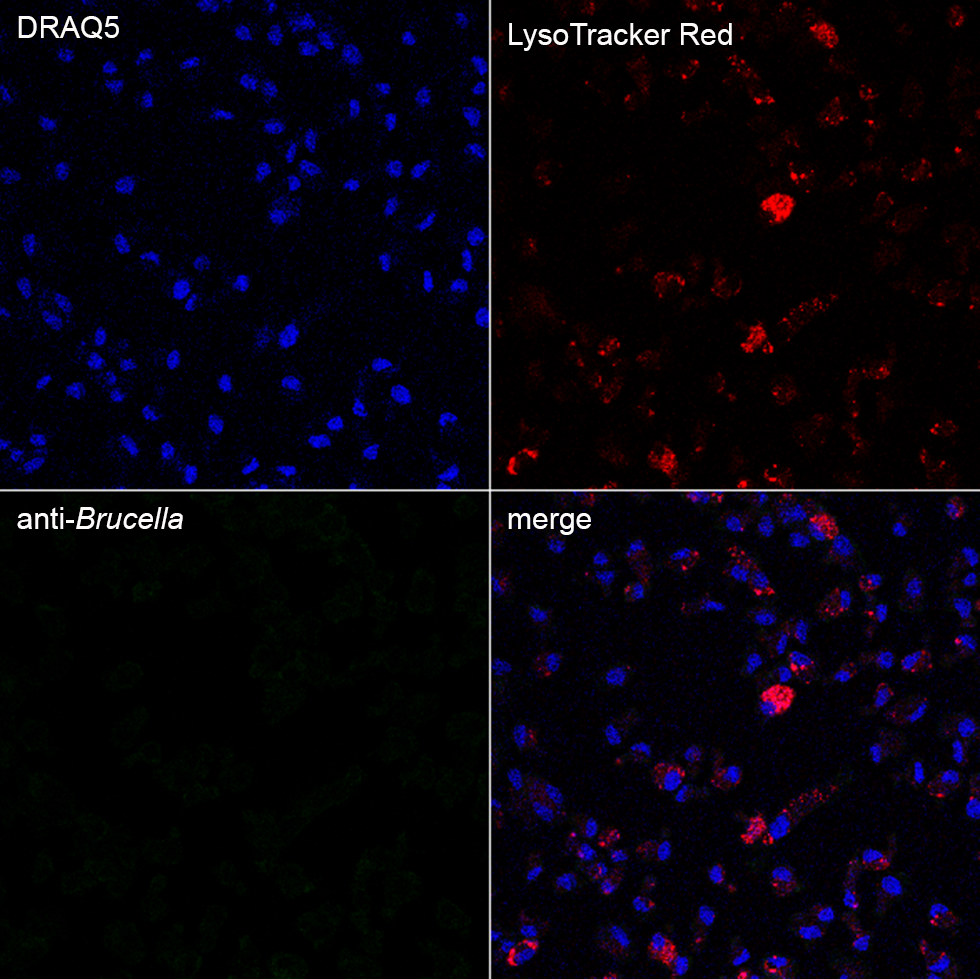

Supplement: Figure S3 — Hooded seal alveolar macrophages were cultured in 12 well plates containing glass coverslips for 5–7 days. Cells were incubated with LysoTracker Red (red) for 1 h before fixed and immune labeled with anti-Brucella antibody 1:100 (green). DRAQ5 was used for visualization of the nuclei (blue). Confocal microscopy revealed no unspecific binding of anti-Brucella antibody in non-infected cells. (TIF) [file pone.0070186.s006.tif]
